# Recent HPV self-sampling use for cervical cancer screening in Latin America and Caribbean: a systematic review

**DOI:** 10.3389/fonc.2022.948471

**Published:** 2022-10-19

**Authors:** Camila B. Dartibale, Gabriela de C. Prado, Lucimara R. Carobeli, Lyvia E. de F. Meirelles, Gabrielle M. Z. F. Damke, Edilson Damke, Fabrício Morelli, Raquel P. Souza, Group PREVENT YOURSELF, Vânia R. S. da Silva, Marcia E. L. Consolaro

**Affiliations:** Laboratory of Clinical Cytology and Infecções Sexualmente Transmissíveis (ISTs), Department of Clinical Analysis and Biomedicine, State University of Maringá, Maringá, Brazil

**Keywords:** cervical cancer, screening, self-sampling, HPV, Latin America, Caribbean

## Abstract

**Objective:**

Cervical cancer is one of the deadliest cancers among women in Latin America and Caribbean (LAC), where most of the countries have not been successful in implementing population-level cytology-based screening programs. An increasing body of evidence supports the validity of self-sampling as an alternative to clinician collection for primary Human *papillomavirus* (HPV) screening. Therefore, this work aims to summarize recent HPV self-sampling approaches in LAC.

**Method:**

We performed a systematic review to identify studies focused on “Self-sampling”, and “Human Papillomavirus DNA test” and “Latin America” in PubMed, Embase, Web of Science, Cochrane library and SCOPUS databases for publications dating between 01 January 2017 and 15 March 2022 based on the Preferred Reporting Items for systematic reviews and meta-analysis (PRISMA) statement. Additionally, the references of the articles were carefully reviewed.

**Results:**

Of the 97 records selected, 20 studies including 163,787 participants, with sample sizes for individual studies ranging from 24 to 147,590 were included in this review. Studies were conducted in 10 LAC countries (18.5%), most with upper medium-income economies (70%). The range of age was 18 to ≥65 years. The vast majority of the studies (85%) addressed the HPV self-sampling strategy for primary cervical cancer screening with overall success for all women including under/never screened and those from special populations (rural, indigenous and gender minorities). Women generally found HPV self-sampling highly acceptable regardless of age, setting of collection, target population or country of residence.

**Conclusions:**

HPV self-sampling is a promising strategy to overcome the multiple barriers to cervical cancer screening in LAC settings and increasing attendance in underscreened women in countries/territories with well-established screening programs. Furthermore, this strategy is useful even in LAC countries/territories without organized cervical cancer screening and in special populations such as indigenous, rural and transgender women. Therefore, the information generated by the recent initiatives for HPV self-sampling approach in LAC can be beneficial for decision-making in both new and existing programs in the region.

## 1 Introduction

Cervical cancer is a largely preventable disease but remains the fourth most common cancer (604,000 new diagnoses) and the fourth leading cause of cancer death (342,000) in women worldwide in 2020 (1). Most of these cases occur in countries where women are not routinely screened or whose programs do not reach quality standards. In well-established successful programs, cases mainly result from women who do not participate in screening (2, 3). Low-and-middle-income countries face the largest burden of this disease, with around 88% of the new global cervical cancer cases and more than 90% of the deaths (4).

Although most Latin America and the Caribbean (LAC) countries and territories today are middle-income economies, there are high heterogeneities across different development indicators (**Supplementary Table 1**). Therefore, recent reports ranked cervical cancer as the third most common cancer diagnosed in the LAC region (5), with considerable variations in incidence and mortality between countries/territories. Cervical cancer remains the leading cause of female cancer in 16.2% of the LAC countries/territories with estimated cancer data available (6). For 2020, it was estimated 56,439 new cervical cancer cases and 31,582 cancer deaths in LAC, with the incidence ranging from 7.2 cases/100,000 women in Martinique to 36.6 cases/100,000 women in Bolivia in (**Supplementary Table 1**). If current trends in incidence and mortality as well as in cervical cancer screening programs coverage in LAC continue, around 89% of the 51,500 cervical cancer deaths predicted for the Americas will occur in LAC in 2030 (7). Therefore, decades of Pap-based screening to detect pre-cancerous cervical lesions in a few countries in the region have not had a major impact in reducing cervical cancer incidence and mortality rates, which are still high across LAC (3, 5–9). There are several factors contributing to this lack of impact: suboptimal sensitivity of the Pap test; the need to perform a pelvic evaluation to collect the cervical sample for Pap test, which could be a significant limiting factor in populations that do not accept such pelvic examinations for cultural reasons; uneven allocation of resources; variable infrastructure and service availability; limited number of population-based cancer registries; scarce distribution of public health centers, which is even more evident in rural areas far from the large urban centers; and weakness of the programs and their inability to perform proper follow-up and treatment of women with positive screening results (3, 8, 9). Taken together, these difficulties result in a scenario of unequal care provided to cancer-affected individuals.

The limitations inherent to Pap tests prompted the development of new screening technologies: tests to detect the presence of Human *Papillomavirus* (HPV) DNA (8). HPV DNA tests have proven to be more sensitive, reproducible and to allow for safer extended screening intervals than conventional cytology or visual inspection with acetic acid (VIA) (10, 11). HPV testing is less dependent on operator expertise than Pap or VIA, making it more suitable for resource-constrained settings. Furthermore, HPV testing can be performed on vaginal samples collected by the woman herself, known as self-sampling. Self-sampling is a safe and easy approach, increasing the opportunities of reaching women that otherwise would not participate in a clinician-based screening or facilitate their access to a screening test (12). Self-sampling is highly acceptable in terms of easy use, convenience, privacy and physical and emotional comfort, in both high- and low and middle-income countries (13). In addition, comparable diagnostic accuracy has also been confirmed for cervical intraepithelial neoplasia grade two or worse of self-collected and clinician collected samples (14–16). Consequently, the WHO now recommends primary HPV based screening and includes self-sampling among the recently published guidelines on self-intervention for health and as part of the cervical cancer screening guidelines (12). The International Agency for Research on Cancer update of the efficacy and effectiveness of cervical cancer screening methods also supports this statement (17).

In recent years, more HPV DNA tests became available and the prices dropped significantly, making possible for eight LAC countries/territories to pilot the introduction of these technologies and more recently, twelve introduced these tests in population-based programs (**Supplementary Table 1**). Therefore, the present systematic review was conducted to summarize the main recent experiences of the HPV self-sampling approach in LAC countries and territories in a context in which an increasing number of countries/territories are switching to virological testing.

## 2 Methods

We conducted this systematic review in accordance to the Preferred Reporting Items for Systematic Reviews and Meta-Analyses (PRISMA) guidelines (18, 19) focusing on the use of the self-sampling approach in LAC countries and territories with or without primary HPV-based screening.

### 2.1 Study definitions

We defined HPV self-sampling as a process in which a patient who wants to screen for HPV infection uses a kit to collect a vaginal sample and send it for analysis by a laboratory. We only included articles that focused on vaginal samples given our interest in cervical cancer. Collection devices include brush, swab and tampon and may occur in any setting (eg, home, community and clinic). We defined HPV clinician sampling as any sampling method where a clinician or other healthcare provider obtains the vaginal sample with speculum.

Additionally, we grouped LAC countries/territories based on the Human Development Index (HDI) using the 2021 World Bank’s classification which economies are currently divided into low, lower-middle, upper-middle and high income economies. Income is measured using gross national income (GNI) per capita, in U.S. dollars, converted from local currency using the *World Bank Atlas* method. Estimates of GNI are obtained from economists in the World Bank country units and the size of the population is estimated by World Bank demographers from a variety of sources, including the UN’s biennial *World Population Prospects*. For the current 2022 fiscal year, low-income economies are defined as those with a GNI per capita of $1,045 or less in 2020; lower middle-income economies are those with a GNI per capita between $1,046 and $4,095; upper middle-income economies are those with a GNI per capita between $4,096 and $12,695; high-income economies are those with a GNI per capita of $12,696 or more (20).

Finally, we classified the self-sampling studies in LAC into two modalities: 1) Pilot studies: those that were carried out as a government initiative in their local, regional or national programs or guidelines to cervical cancer screening; 2) Independent studies: research studies carried out independently of governmental initiatives.

### 2.2 Inclusion criteria

Studies were eligible for inclusion if they met the following criteria (1): included participants of LAC who performed or evaluated vaginal self-sampling for HPV DNA testing (2); original publications in English and Spanish languages and (3) published in a peer-reviewed journal in the last five years (01 January 2017 and 15 March 2022). Both qualitative and quantitative studies were included.

### 2.3 Search strategy and screening process

We performed a systematic review to identify studies focused on “Self-sampling”, and “Human Papillomavirus DNA test”, “Latin America” and “Caribbean” in PubMed, Embase, Web of Science, Cochrane library and SCOPUS databases for publications dating between 01 January 2017 and 15 March 2022 based on the 2020 PRISMA statement (19). To identify original publications in English and Spanish languages, researchers (Group PREVENT YOURSELF, CBD, GCP, LRC, LEFM, GMZFD, ED, FM, RPS) performed independent searches using various combinations of descriptors in PubMed/Embase or as a topic in WOK (“Self Care” OR “Self-Testing” OR “House Calls” AND “Self Care” OR “Self-Testing” OR “House Calls” AND “Papillomavirus Infections” OR “Papillomaviridae” OR “Alphapapillomavirus” OR “Human Papillomavirus DNA Tests” AND “Caribbean Region” OR “Central America” OR “South America” OR “Latin America”).

Titles and abstracts were carefully selected to ensure publication originality and quantitative and qualitative consensus. The initially selected studies had to fit the following two criteria: the first criteria included original epidemiological and clinical studies involving HPV self-sampling for HPV DNA detection in LAC. The second criteria was to exclude duplicate studies, review studies, letters to editor and books. After consensus, the papers most closely related to the theme descriptors were selected. Then, the full-text articles were randomly distributed to all the investigators (Group PREVENT YOURSELF, CBD, GCP, LRC, LEFM, GMZFD, ED, FM, RPS, VRSS, MELC) who acted as independent evaluators in charge of the inclusion of articles in the final cohort, for data extraction. Any disagreement was resolved by discussing with the senior author (MELC). To increase the sensitivity of the search, the references of the original articles were carefully reviewed for recovery articles that could be additionally utilized in this review. To ensure that all relevant data from each paper were included in the review, a final consensus was achieved following an additional examination of the full texts by two individual experts (VRSS, MELC).

### 2.4 Data extraction and analysis

Two reviewers independently used a standardized data abstraction form to capture information on location of study, HDI, study characteristics and type, study population, sample size and results for HPV DNA self-sampling from each study. Differences in data abstraction were resolved through consensus by a third reviewer as needed.

Data was analyzed and then processed using Excel™ with the aim to display all relevant information in an organized manner.

## 3 Results

### 3.1 Selection of studies

**Figure 1:**
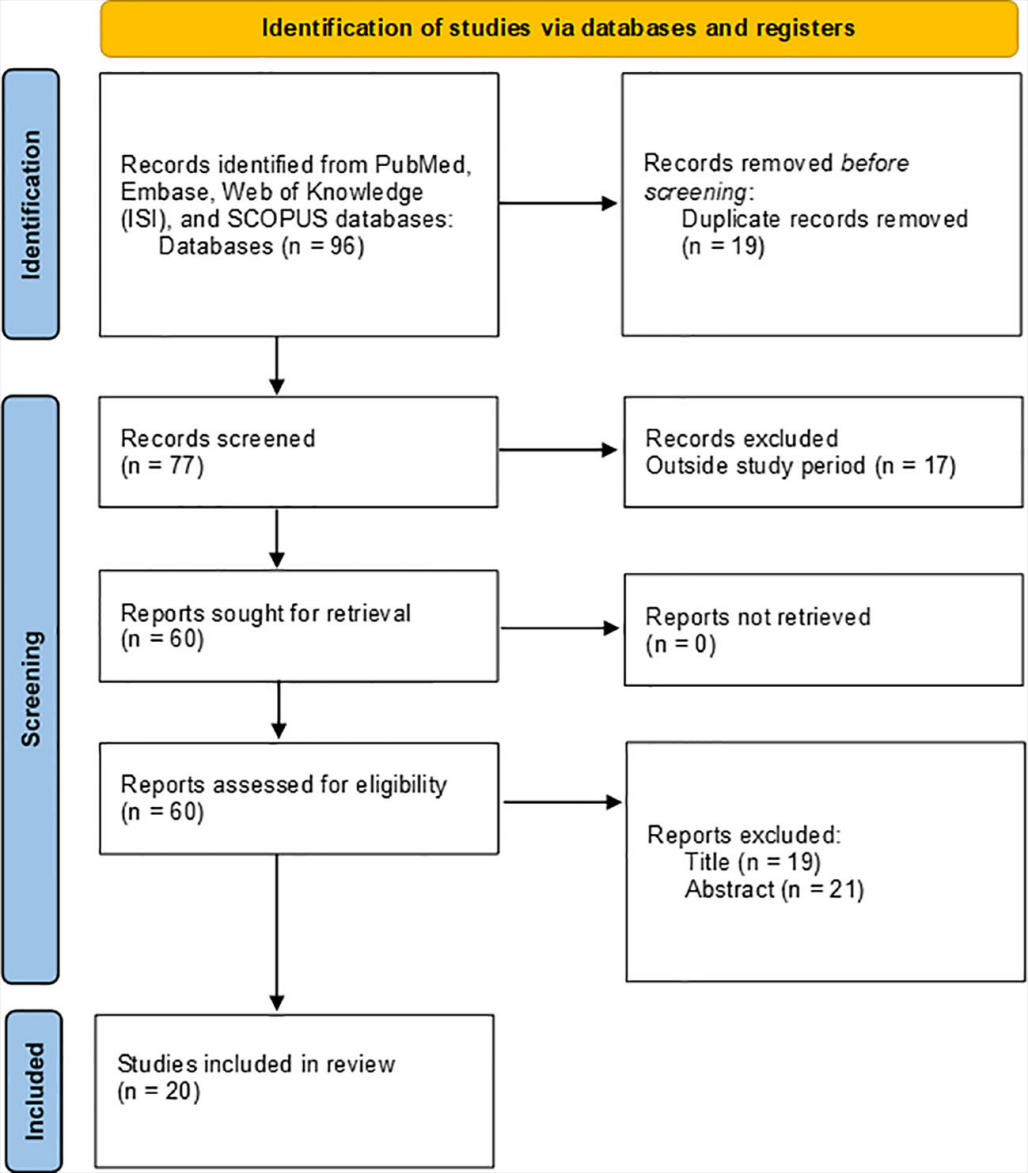
PRISMA flow diagram used in this systematic review.

We selected 85 records *via* electronic databases and references of papers, with 11 additional citations reviewed from references listed in prior reviews, including studies and hand-searches. Of the 96 records, 19 were excluded because they were duplicated and 17 because they were outside the period determined for the review. Following, 40 articles were omitted after reviewing the title and abstracts. Finally, 20 studies involving the use of vaginal self-sampling for HPV DNA detection in LAC in the last five years were included in this systematic review (**Figure 1** **-** PRISMA flow diagram).

### 3.2 Characteristics of the included studies

#### 3.2.1 Overall characteristics

**Table 1** presents summary characteristics of the 20 included studies. Details of the included studies are presented in **Table 2**. The 20 studies included at least 163,787 participants, the sample sizes for individual studies ranged from 24 to 147,590 participants and 45% were published in 2020. All included studies were conducted in 10 LAC countries/territories which in only two (Argentina and El Salvador) the national cervical cancer screening program recommended the HPV DNA test. These 10 LAC countries are in South America (50%) followed by Central America (40%) and North America (10%) (**Table 2**). No studies from the Caribbean region were included in this systematic literature review.

**Table 1:** Summary description of included studies.

Characteristic	Articles*
Region	
North America: Mexico (21–23).	**3**
South America: Argentina (24–27), Bolívia (28–30), Brazil (31–33), Colombia (34), Peru (35).	**12**
Central America: El Salvador (36, 37), Guatemala (38, 39), Guatemala, Nicaragua and Honduras (40).	**5**
Populations (not mutually exclusive)	
Women from the general population (23, 28–30, 34, 40).	**6**
Women from the rural areas (21, 33, 38).	**3**
Neverscreened or underscreened (24, 31, 32, 36)	**4**
Indigenous women (21, 38, 39)	**3**
Women HPV+ by self-sampling (25–27)	**3**
College students (35)	**1**
Sexual and gender minorities (37)	**1**
Women with previous diagnosis of dysplasia (22)	**1**
Study design	
Qualitative (21, 22, 25)	**3**
Quantitative (23, 24, 26–40)	**17**
Specimen collection devices	
Swab (21, 29, 30, 38, 39)	**5**
Brush (22–24, 26, 31–37, 40)	**12**
Multiple devices (28)	**1**
Unspecified/Not used (25, 27)	**2**
Setting for self-sampling	
Clinic (22, 23, 30, 35, 37)	**5**
Home (24, 25, 31, 32, 36, 38, 39)	**7**
Community setting (28)	**1**
Multiple Settings (21, 26, 27, 29, 33, 34, 40)	**7**

*The number of studies within each category is not mutually exclusive.

HPV, Human papillomavirus.

HPV+, Positive HPV test.

**Table 2:** Characteristics of included studies.

First author, year	Country/Territory	Location	Income	HPV test	Self-sampling use*	Self-sampling device	Setting	Target population	Age (years)	Sampling size	Study design	Main findings of the study
Arrossi, 2017 (24)	Argentina	South America	Upper middle-	In national program	Pilot study	Brush	Home	Underscreened women	30+	2983	Quantitative: databases analysis based on Health System Framwork	HPV self-sampling offered by CHWs at home visits can be adequately scaled-up in programmatic conditions to increase screening of hard-to-reach women.
Arrossi, 2019 (26)	Argentina	South America	Upper middle-	In national program	Pilot study	Brush	Multiple settings	Special population (HPV+)	30+	4865	Quantitative: multi-component mobile health (mHealth) intervention to increase adherence to triage	Expected to improve follow-up results for women with HPV+ self-sampling testing.
Antelo, 2020 (25)	Argentina	South America	Upper middle-	In national program	Pilot study	NU	Home	Special population (HPV+)	30+	48	Qualitative: use of SMS to be tested in the trial.	HPV+ women by self-sampling preferred not receive negative results *via* SMS because they believed that the communication between them and the health professionals during the delivery of the results should be prioritized.
Paolino, 2020 (27)	Argentina	South America	Upper middle-	In national program	Pilot study	NU	Multiple settings	Special population (HPV+)	30+	2389	Quantitative: databases analysis based on Public Health System	The adherence of HPV+ women who performed self-sampling to triage test (cytology) at 18 months was low (42.9%).
Surriabre, 2017 (28)	Bolivia	South America	Lower middle-	Pilot study	Research	Multiple devices	Community	All women	25-59	222	Quantitative: study evaluating the possibility of introducing self-sampling	Most women preferred self-sampling over clinician-sampling for cervical cancer screening.
Allende, 2019 (29)	Bolivia	South America	Lower middle-	Pilot study	Research	Swab	Multiple settings	All women	25-64	1123	Quantitative: cross sectional study	Despite greater acceptance of the HPV self-sampling, women kept greater confidence in the screening performed by the gynecologist.
Allende, 2020 (30)	Bolivia	South America	Lower middle-	Pilot study	Pilot study	Swab	Clinic	All women	25-64	362	Quantitative: cross sectional study	Self-sampling could overcome sociocultural barriers to cervical cancer screening.
Torres, 2018 (33)	Brazil	South America	Upper middle-	Pilot study	Pilot study	Brush	Multiple settings	Special population (Rural)	18+	412	Quantitative: cross sectional study	Self-sampling had a high level of acceptance with 80% of women preferring this mode of collection than by a health professional.
Castle, 2019 (31)	Brazil	South America	Upper middle-	Pilot study	Pilot study	Brush	Home	Never/Underscreened women	25-65	483	Quantitative: study evaluating the preference and adherence to self-sampling	Self-sampling is a promising strategy for un/under-screened women who are recalcitrant or unable to undergo clinic-based cervical screening.
Pantano, 2021 (32)	Brazil	South America	Upper middle-	Pilot study	Pilot study	Brush	Home	Never/Underscreened women	30+	355	Quantitative: study evaluating the acceptabilityto self-sampling	Self-sampling is an adequate strategy to improve the effectiveness of the cervical cancer program by increasing screening in a high-risk group.
Torrado-García, 2020 (34)	Colombia	South America	Upper middle-	In national program	Pilot study	Brush	Multiple settings	All women	35-65	423	Quantitative: cross sectional study	Women living in low-income households preferred the self-sampling procedure (98% of acceptability).
Manrique-Hinojosa, 2018 (35)	Peru	South America	Upper middle-	In national program	Research	Brush	Clinic	Special population (College students)	18-30	221	Quantitative: transversal study	The frequency of high- risk HPV was greater in the group through the self-sampling in comparison with previous national investigations.
Laskow, 2017 (36)	El Salvador	Central America	Lower middle-	In national program	Pilot study	Brush	Home	Underscreened women	30-59	60	Quantitative: self-sampling feasibility and acceptability	For a majority of non-attenders women, CHWs-based self-sampling was an acceptable way to participate in a cervical cancer screening program.
Maza, 2020 (37)	El Salvador	Central America	Lower middle-	In national program	Pilot study	Brush	Clinic	Special population (Transgender men)	19-55	24	Quantitative: feasibility of using self-sampling	HPV self-sampling was accepted by the majority of participants.
Gottschlich, 2017 (39)	Guatemala.	Central America	Upper middle-	Pilot study	Pilot study	Swab	Home	Special population (indigenous)	25-54	202	Quantitative: cross sectional study	HPV self-sampling samples were well accepted by indigenous communities.
Murchland, 2019 (38)	Guatemala.	Central America	Upper middle-	Pilot study	Pilot study	Swab	Home	Special population (Indigenous and rural)	18-60	956	Quantitative: self-sampling acceptability	HPV self-sampling was highly acceptable in rural and indigenous communities.
Holme, 2020 (40)	Guatemala, Honduras, and Nicaragua	Central America	Upper middle-, Lower middle- and Lower middle-	Pilot study	Pilot study	Brush	Multiple settings	All women	30+	147590	Quantitative: self-sampling introduction in public health centers	HPV testing, including self-sampling, was acceptable and feasible to implement for a large volume of women across the three countries and achieved a high coverage between screened women.
Allen-Leigh, 2017 (21)	Mexico	North America	Upper middle-	In national program	Pilot study	Swab	Multiple settings	Special population (Indigenous and rural)	20+	503	Qualitative: self-sampling barriers	Low-income, indigenous women residing in rural, underserved areas found a number of advantages of HPV self-sampled tests.
Flores, 2021 (23)	Mexico	North America	Upper middle-	In national program	Research	Brush	Clinic	All women	30-65	505	Quantitative: performance and acceptability of self-sampling	Self-sampling was well accepted among study participants.
Rodrıguez, 2021 (22)	Mexico	North America	Upper middle-	In national program	Research	Brush	Clinic	Special population (previous dysplasia)	NI	61	Qualitative: attitudes and acceptability of self-sampling	Women reported high acceptability for self-sampling and positive attitudes toward HPV diagnostic procedures.

NI, Not informed; NU, Not used.

*, Pilot study- those that were linked to a government initiative in their local, regional or national programs or guidelines; Research studies- those that were not linked to a governmental initiative.

CHWs, community health workers.

HPV, Human papillomavirus.

HPV+, Positive HPV test.

SMS, Short Message Service.

#### 3.2.2 Participants characteristics

Participants ranged in age from 18+ with the 40% being 30+. However, many studies do not specify the maximum age of the participants included (21, 24–27, 32, 33, 40). Four studies specifically targeted women who were under/never screened for cervical cancer (24, 31, 32, 36). The remaining studies selected participants from specific subgroups or vulnerable populations, including women from rural areas (21, 33, 38), indigenous (21, 38, 39), gender minorities (transmales) (37), college students (35), others as HPV+ women by self-sampling (25–27) and with previous diagnosis of dysplasia (22).

#### 3.2.3 Studies design

Most included studies were quantitative (23, 24, 26–40). These studies examined a wide range of end users, including under/never screened (24, 31, 32, 36) and vulnerable subpopulations such as indigenous women (21, 38, 39), women from rural areas (21, 33, 38) and transgender men (37). Of these studies, 50% included women above the age 30 followed by 37.5% of women above 25. Most quantitative studies (75%) focused on end users in upper-middle-income countries, while only 25% were conducted in lower middle-income countries.

In general, in these quantitative studies, self-sampling has great acceptability for all women (23, 28, 29, 33, 34, 40), for women from special populations (21, 37–39) and in never/under screened women (24, 31, 32, 36). Furthermore, the self-sampling strategy was ratified as an important tool for increased coverage to cervical cancer screening in several of these studies (23, 24, 26, 28, 30–34, 36–40). In studies evaluating women’s preference for the method of collection, most preferred self-sampling over clinician-sampling for cervical cancer screening (28, 31, 33, 34).

Three studies employed a qualitative design method that included in-depth interviews and focus group discussions, to explore women’s acceptability and preferences related to HPV self-sampling (21, 22, 25). Of these, two studies were conducted in North America (21, 22) and 1 in South America (25), all in upper middle-income countries; all focused on special populations such as indigenous and rural women (21), HPV+ women by self-sampling (25) and women with a previous diagnosis of dysplasia (22). Specifically, Antelo et al. (25) analised the content of the SMS in the trial among women with HPV+ self-sampling tests. The data showed that SMS is accepted when notifying these women, but it should not replace the delivery of results in doctor-patient encounters. Allen-Leigh et al. (21) studied the barriers to use of self-sampled HPV testing and cytology among low-income, indigenous women residing in rural areas. They showed that these women found a number of advantages of HPV self-sampled tests. Finally, Rodriguez et al. (22) assessed attitudes and acceptability of self-sampling among women with a previous diagnosis of cervical dysplasia and showed high acceptability.

#### 3.2.4 Self-sampling strategy for cervical cancer screening

The vast majority of studies (85%) addressed the HPV self-sampling strategy for primary cervical cancer screening. Overall, in these studies, the strategy of self-sampling as a primary screening for cervical cancer was successful for both all women and those from special populations.

On the other hand, 15% of the studies evaluated interventions to increase triage adherence among women with HPV+ self-sampled tests (25–27). However, the results were varied, not allowing to conclude the real impact on the follow-up of these women.

#### 3.2.5 Settings and devices for self-sampling

End users self-sampled from their homes (35%) (24, 25, 31, 32, 36, 38, 39), in multiple settings (35%) (21, 26, 27, 29, 33, 34, 40), in clinics (25%) (22, 23, 30, 35, 37) and in the community (28). In general, the self-sampling strategy was well accepted in the different settings in which it was offered.

Among the studies that used one type of device for self-sampling, the brush was the most used (70.6%) (22–24, 26, 31–37, 40), followed by swab (29.4%) (21, 29, 30, 38, 39) and both were well accepted.

#### 3.2.6 Geographic region and income

The vast majority of the studies were conducted in South America (60%), followed by Central America (25%) and North America (15%). No studies from the Caribbean region were found that met our inclusion criteria.

**Figure 2:**
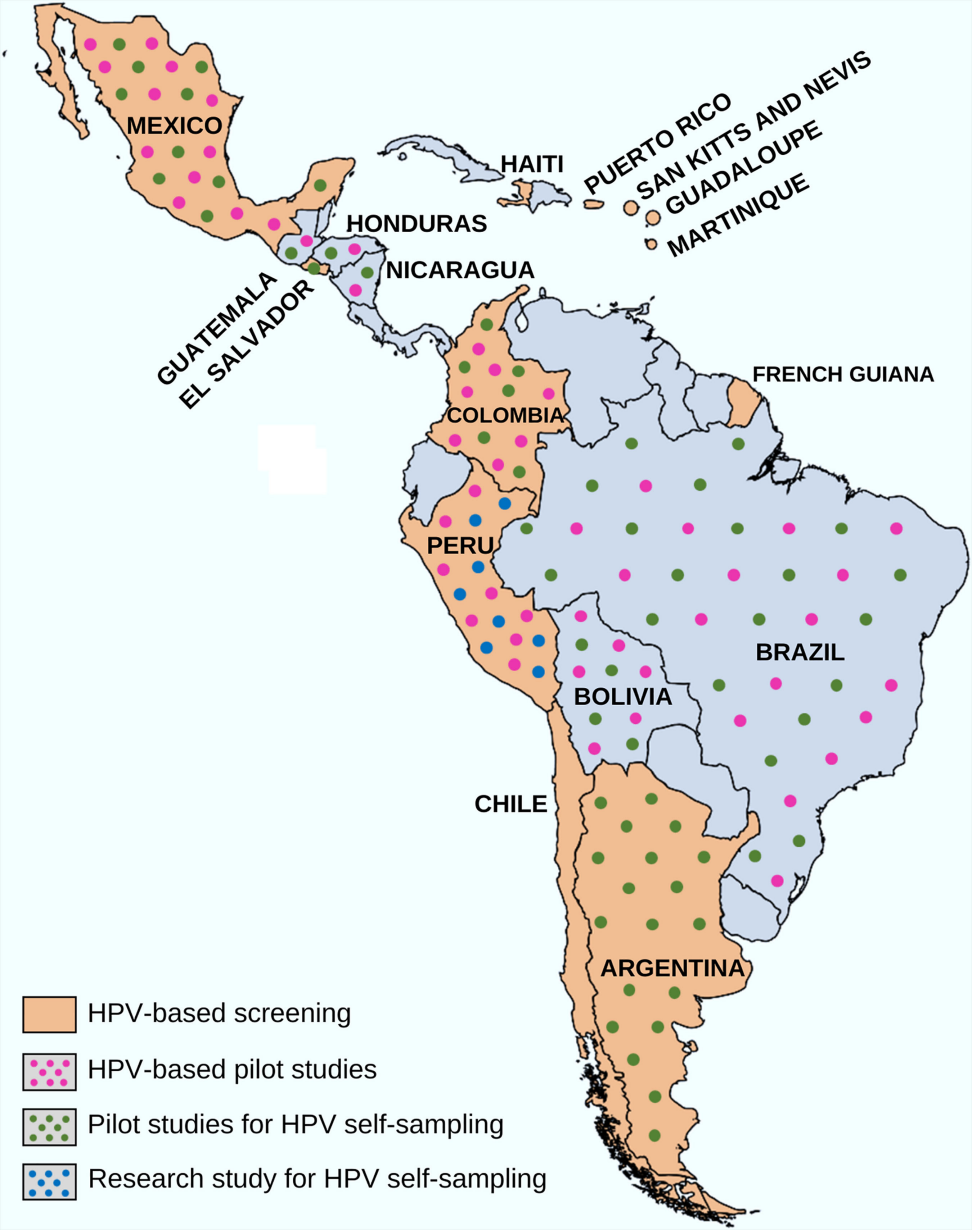
Recent HPV self-sampling approach in Latin America and Caribbean countries and territories.

Specifically in South America, the country with more studies was Argentina, in Central America was Guatemala and in North America was Mexico. Among the 20 studies, 15 (75%) introduced self-sampling as a pilot in their local, regional or national programs or guidelines to cervical cancer screening including Argentina (n = 4), Bolivia (n = 1), Brazil (n =3), Colombia (n = 1), El Salvador (n = 2), Guatemala (n = 2), Honduras, Guatemala and Nicaragua (n = 1), and Mexico (n = 1). Five additional studies were not linked to programs or guidelines. These studies were carried out in Bolivia (n = 2), Peru (n =1) and Mexico (n = 2) (**Figure 2**). Despite being the result of independent research, these studies can support the decision whether to include self-sampling in their countries’ screening guidelines for all women (Bolivia and Mexico) and for special populations (Peru and Mexico).

Furthermore, among the 20 studies included, 14 were performed in upper-middle, 5 in low-middle and 1 in both upper-middle and low-middle income. Of the included participants, around 91% were from low-middle-income countries.

#### 3.2.7 Sexual and gender minorities

Only one study conducted in El Salvador examined preferences among sexual and gender minorities (37). The results showed that among transmales who had undergone self-sampling for HPV, 95.6% expressed a preference for self-sampling and willingness to self-sample in the future.

## 4 Discussion

The present work summarizes the current approaches to cervical cancer screening by HPV self-sampling in LAC, in a context in which an increasing number of countries/territories are switching to HPV testing. Overall, this systematic review contains twenty eligible studies involving at least 163,787 participants. The data from these studies are summarized in **Tables 1** , **2**. The vast majority of studies (85%) addressed the HPV self-sampling strategy for primary cervical cancer screening and overall, it was successful for all women including under/never screened and those from special populations (rural, indigenous and gender minorities).

Currently, twelve of the 39 LAC countries/territories (30.8%) introduced HPV testing as a primary screening method for cervical cancer in population-based programs (Argentina, Colombia, Chile, El Salvador, French Guiana, Guadeloupe, Haiti, Mexico, Martinique, Peru, Puerto Rico, and San kitts and Nevis). In addition, at least five countries/territories have developed pilot studies to use the HPV test as a primary screening for cervical cancer (Bolivia, Brazil, Guatemala, Honduras, and Nicaragua) (**Supplementary Tables 1**). Thus, LAC is moving toward the change to HPV testing for cervical cancer screening, with the endorsement of several regional experiences that resulted in increased coverage and better detection of precancerous lesions using HPV tests. This represents a great opportunity to use the HPV self-sampling for primary cervical cancer screening in the region. Indeed, the recent use of HPV self-sampling as a pilot study (linked to a government initiative in their local, regional or national programs or guidelines) was performed in 9 countries (Argentina, Bolivia, Brazil, Colombia, El Salvador, Guatemala, Honduras, Nicaragua and Mexico) at the time of this review. The HPV self-sampling approach was conducted as research study (not linked to a governmental initiative) in Peru (**Table 2**). Additionally, no studies from the Caribbean region were found that met our inclusion criteria. This data may suggest that the HPV self-sampling strategy has recently been even less explored for cervical cancer screening in the Caribbean region than in other LAC regions. This hypothesis is reinforced by cervical cancer estimates for the year 2018 in LAC: incidence rates lower in Central America (13.0 per 100,000) than in South America (15.2) and the Caribbean (15.5), and mortality rates higher in the Caribbean (8.5) than in South America (7.1) and Central America (7.0) (41). However, it should be considered that the COVID-19 pandemic may have influenced initiatives to use self-sampling for HPV testing in LAC by changing health systems priorities. Possibly, only in the post-pandemic period will the real impact of the COVID-19 pandemic on LAC approaches to cervical cancer screening by HPV self-sampling be determined.

Barriers to cervical cancer control in LAC include uneven allocation of resources, variable infrastructure and service availability, limited number of population-based cancer registries and scarce distribution of public health centers, which is even more evident in rural areas far from the large urban centers. Taken together, these difficulties result in a scenario of unequal care provided to cancer affected individuals (9). However, at least part of these barriers can be overcome with the introduction of HPV self-sampling. Still, there are several opportunities in LAC that are making the HPV self-sampling approach more feasible and faster than in other word regions. The first opportunity is that most LAC countries/territories (around 72%) already have primary cervical cancer screening programs funded and led by the national government (**Supplementary Table 1**); this means that countries already have these activities in their national budget, facilitating the process for reallocating some of that funding for HPV testing and self-sampling activities. Other advantages of having such programs already in place is to implement the culture of screening for cervical cancer among women and providers. Also, women will understand the value of prevention and will adopt new options such as self-collecting a vaginal sample. In addition, several LAC countries/territories have started free vaccination programs aimed at girls between the ages of 9 and 13 years in schools and health facilities or health centers (42). Although vaccination coverage is very low (43), this is an important initiative in the region, as both primary prevention (vaccination) and secondary prevention (screening) are needed to resolve the burden of cervical cancer in LAC.

Our findings still show that among the studies that addressed the HPV self-sampling strategy for primary cervical cancer screening, there were many differences between various aspects such as device type, materials and HPV DNA test used, number of participants and target population. Regarding the setting for the self-sampling, only 35% of the studies were conducted exclusively at the participants’ homes, which makes it difficult to conclude about the places preferences of the women included. There are few governments HPV self-sampling initiatives from previous periods, as in the case of Argentina. Still, there are few initiatives integrating self-sampling studies between different countries in the region, as in the case of the joint study of Guatemala, Honduras and Nicaragua. Finally, no studies with HPV self-sampling have been conducted in low-income economies of LAC and in the Caribbean. Therefore, our data underscored the need for additional research on self-sampling in LAC. First, we found very few studies from LAC evaluating validity and economic viability in the region. More studies are required across different LAC countries/territories to confirm self-sampling validity and to ensure reliability. In addition, our search found published studies on self-sampling from only 10 of the 54 LAC countries/territories in the past 5 years. Further, only five of the ten LAC countries/territories with the highest rates of cervical cancer globally were represented, highlighting the dearth of research in this area. More studies are needed to improve the applicability and generalizability of results across different LAC contexts.

Despite its potential benefits, the implementation of HPV self-sampling faces some challenges, including training healthcare workers to explain the self-sampling procedure adequately to participating women, transportation of the collected specimens, laboratory technical differences between cervical and vaginal samples processing and finally, skilled clinicians to manage and follow-up positive women (44–46). Regarding follow-up, few of the studies included in this review focused on this theme and used different strategies for the follow-up of HPV+ women by self-sampling (25–27). At the same time, the several opportunities in LAC that can make the process more feasible and faster than in other regions of the world are mainly: most LAC countries/territories already have screening programs funded by their national governments, several countries in the region are already implementing HPV testing and there is a regional pooled procurement mechanism that could facilitate the purchase of HPV tests at an accessible price. Additionally, the experience from the different LAC countries has created rich information about the barriers and requirements for implementing HPV self-sampling primary screening at large scale in the region.

In summary, the HPV self-sampling approach is now considered a key pillar to reach the WHO cervical cancer elimination target (12). Furthermore, the results of recent studies show that HPV self-sampling is a promising strategy to overcome the multiple barriers to cervical cancer screening in LAC settings and increasing attendance in underscreened women in countries/territories with well-established screening programs. Additionally, this strategy is useful even in LAC countries/territories without organized cervical cancer screening and in special populations such as indigenous, rural and transgender women. Thus, the information generated by the recent initiatives for HPV self-sampling approach in LAC can be beneficial for decision-making in both new and existing programs in the region.

## Limitations

To the best of our knowledge, this is the first study to systematically review the self-sampling approach in LAC countries/territories as a pilot study linked to government initiatives or independent studies, which are those not linked to government initiatives. Findings from this review should be viewed in light of its limitations. We did not include conference abstracts, books, reviews and articles published in other languages than English or Spanish in this review, so our findings may not fully represent the full body of literature on HPV self-sampling in LAC. Also, in the current COVID-19 pandemic scenario, the opportunity to renew and make cervical cancer screening more resilient, highlighting the advantages of risk-based management, HPV-based screening and in particular, the use of HPV self-sampling has been discussed (47). On the other hand, economic factors and varying healthcare priorities due to the COVID-19 may have limited studies and the implementation of HPV-based screening in LAC and consequently self-sampling as well.

## Group members of Group PREVENT YOURSELF

Débora M. G. Sant’Ana^1^, Sandra M. Pelloso^1^, Isabel C. Scarinci-Searles^2^, Valquiria C. A. Martins^3^, Cláudia M. Carneiro^4^, Rita G. Amaral^5^, Janaina C. O. C. Freitas^6^

^1^State University of Maringá, Maringá, PR, Brazil

^2^University of Alabama at Birmingham, Birmingham, AL, USA

^3^Amazonas Oncology Control Center Foundation, Manaus, AM, Brazil

^4^Federal University of Ouro Preto, Ouro Preto, MG, Brazil

^5^Federal University of Goiás, Goiânia, GO, Brazil

^6^Federal University of Rio Grande do Norte, Natal, RN, Brazil

## Data availability statement

The original contributions presented in the study are included in the article/**Supplementary Material**. Further inquiries can be directed to the corresponding author.

## Author contributions

CD, Group PREVENT YOURSELF and MC, conceptualization, investigation, writing-original draft, writing - review and editing. GP, LC, LF, GF, ED, FM, RS: literature search, Study selection, Data Curation, Visualization, Writing - Original Draft. VS: literature search, study selection, Data Curation and Writing - Review & Editing. All authors contributed to the article and approved the submitted version.

## Funding

This work was supported by the Departamento de Ciência e Tecnologia da Secretaria de Ciência, Tecnologia e Insumos Estratégicos do Ministério da Saúde/BR (Decit/SCTIE/MS).

## Conflict of interest

The authors declare that the research was conducted in the absence of any commercial or financial relationships that could be construed as a potential conflict of interest.

## Publisher’s note

All claims expressed in this article are solely those of the authors and do not necessarily represent those of their affiliated organizations, or those of the publisher, the editors and the reviewers. Any product that may be evaluated in this article, or claim that may be made by its manufacturer, is not guaranteed or endorsed by the publisher.
