# Supplementary material for: Recent HPV self-sampling use for cervical cancer screening in Latin America and Caribbean: a systematic review
Source: Front Oncol. 2022 Oct 19;12:948471. doi: 10.3389/fonc.2022.948471 (PMC9627290; doi:10.3389/fonc.2022.948471)
Supplement: Supplementary file 1 [file Table_1.docx]

Supplementary Material

**Supplementary Table 1. Characteristics of actual cervical cancer incidence and screening in Latin America and Caribbean**

| **Country/Territory** | **Location** | **Income** | **Age-standardized incidence/ 100,000 women (2020)** | **National program** | **Modality of national program** | **HPV DNA test in national program** | **Screening method** | **How is the program** |
| --- | --- | --- | --- | --- | --- | --- | --- | --- |
| Argentina | South America | Upper  middle- | 16.7 | Yes | Organized | Yes | HPV test/Pap test | Age 35-64 years (Pap test), 30+ years (HPV test).  Pap test every 3 years, after 2 consecutive annual negative tests.  HPV test every 3 years. |
| Bolivia | South America | Lower  middle- | 36.6 | Yes | Opportunistic | No | Pap test/VIA | Age 25-64 years.  Pap test every 3 years, after 2 consecutive annual negative tests. |
| Brazil | South America | Upper  middle- | 12.7 | Yes | Opportunistic | No | Pap test | Age 25-64 years.  Pap test every 3 years, after 2 consecutive annual negative tests. |
| Chile | South America | High- | 11.1 | Yes | Organized | Yes (but two guidelines) | Pap test/HPV test | Age 25-64 years (Pap test), 30-64 years (HPV test).  Pap test every 3 years.  HPV test every 5 years. |
| Colombia | South America | Upper  middle- | 14.9 | Yes | Organized | Yes | Pap test/ HPV test/VIA | Age 25-69 years (Pap test), 30-69 years (HPV test), and 30-50 v (VIA).  Pap test every 3 years, after 2 consecutive annual negative tests. HPV test every 5 years. VIA every 3 years. |
| Ecuador | South America | Upper  middle- | 16.0 | Yes | Organized | No | Pap test | Age 35-64 years.  Pap test every 5 years. |
| French Guiana | South America | Middle-income | 22.6 | Yes | Organized | Yes | Pap test/HPV test if ASC-US | Age 25-65 years (Pap test).  Pap test every 3 years, after 2 consecutive annual negative tests. |
| Guyana | South America | Lower  middle- | 29.5 | Yes | Organized | No | VIA | Age 25-49 years. |
| Paraguay | South America | Upper  middle- | 34.1 | Yes | Opportunistic | No | Pap test/VIA | Pap test/VIA every 3 years, after 3 consecutive annual negative tests. |
| Peru | South America | Upper  middle- | 22.2 | Yes | Organized | Yes | HPV test or VIA and Pap test | Age 30-49 years (VIA), 50-65 years (Pap test).  HPV test every 5 years  VIA every 3 years  Pap test every 3 years. |
| Suriname | South America | Upper  middle- | 23.7 | No | NI | No | NI | No National program. |
| Uruguay | South America | High- | 11.7 | Yes | Organized | No | Pap test | Age 21-69 years.  Pap test every 3 years, after 2 consecutive annual negative tests. |
| Venezuela | South America | High- | 22.2 | Yes | Organized | No | Pap test | Age 25-64 years.  Pap test every 3 years. |
| Belize | Central America | Upper  middle- | 19.1 | Yes | Opportunistic | No | Pap test | Age 18-49 years.  Pap test every 3 years, after 3 consecutive annual negative tests. |
| Costa Rica | Central America | Upper  middle- | 11.7 | Yes | Opportunistic | No | Pap test | Age ≥ 20 years.  Pap test every 1 year. |
| El Salvador | Central America | Lower  middle- | 13.1 | Yes | Organized | Yes | HPV test/ Pap test/VIA | Age 30-59 years.  Screening interval: 2 years |
| Guatemala | Central America | Upper  middle- | 20.3 | Yes | Opportunistic | No | Pap test/VIA | Age 25-54 years.  Screening interval: 3 years |
| Honduras | Central America | Lower  middle- | 19.5 | No | Opportunistic | No | Pap test/VIA | Age 30-59 years.  Test every 3 years, after 3 consecutive annual negative tests. |
| Nicaragua | Central America | Lower  middle- | 21.3 | Yes | Opportunistic | No | Pap test/VIA | Age 25-64 (Pap tesy), 30-50 (VIA).  Pap test/VIA every 3 years, after 3 consecutive annual negative tests. |
| Panama | Central America | High- | 14.0 | Yes | Opportunistic | No | Pap test/VIA | Age 20-64 years.  Test every 3 years, after 3 consecutive annual negative tests. |
| Mexico | North America | Upper  middle- | 12.6 | Yes | Opportunistic | Yes | Pap test/ HPV test | Age 25-64 years (Pap test), ≥ 34 years (HPV test.  Pap test every 3 years, after 2 consecutive annual negative tests. |
| Antígua e Barbuda | Bolivarian Republic of The Caribbean | High- | NI | Yes | Opportunistic | Yes | Pap test/ HPV test | Age 21-65 (Pap test), ≥ 30 (HPV test).  Test every 5 years (Pap test) . |
| Bahamas | Bolivarian Republic of The Caribbean | High- | 14.9 | Yes | Opportunistic | No | Pap test | Age 21-59 years.  Pap test every 1 year. |
| Barbados | Bolivarian Republic of The Caribbean | High- | 15.2 | Yes | Opportunistic | No | Pap test | Age 21-65 years. Screening ages (years).  Pap test every 3 years (ages 21-49), 5 years (ages 50-65). |
| Cayman Islands | Bolivarian Republic of The Caribbean | High- | NI | Yes | NI | No | Pap test | First Pap smear within two years of becoming sexually active. If still a virgin at the age of 21, should have her first Pap smear.  Pap test every 2 years. |
| Cuba | Bolivarian Republic of The Caribbean | Upper  middle- | 13.9 | Yes | Opportunistic | No | Pap test | Age 25-64 Years.  Pap test every 3 years. |
| Dominica | Bolivarian Republic of The Caribbean | Upper  middle- | NI | Yes | Opportunistic | No | Pap test | Age 18-65 years. |
| Dominican Republic | Bolivarian Republic of The Caribbean | Upper  middle- | 17.9 | Yes | Opportunistic | No | Pap test | Age 35-64 years.  Pap test every 3 years, after 2 consecutive annual negative tests. |
| Grenada | Bolivarian Republic of The Caribbean | Upper  middle- | NI | No | Opportunistic | No | Pap test | Age ≥ 21 years.  Pap test every 3 years, after 3 consecutive annual negative tests. |
| Guadaloupe | Bolivarian Republic of The Caribbean | Upper  middle- | 8.7 | Yes | Organized | Yes | Pap test/ HPV test if ASC-US | Age 25-64 years.  Pap test every 3 years, after 3 consecutive annual negative tests. |
| Haiti | Bolivarian Republic of The Caribbean | Lower  middle- | 11.6 | Yes | Opportunistic | Yes | HPV test | Age 40-80 years. |
| Jamaica | Bolivarian Republic of The Caribbean | Upper  middle- | 21.6 | Yes | Opportunistic | No | Pap test | Age 25-54 years.  Pap test every 3 years, after 2 consecutive annual negative tests. |
| Martinique | Bolivarian Republic of The Caribbean | Upper  middle- | 7.2 | Yes | Organized | Yes | Pap test/HPV test if ASC-US | Age 25-65 years.  Pap test every 3 years, after 2 consecutive annual negative tests. |
| Puerto Rico | Bolivarian Republic of The Caribbean | High- | 8.0 | Yes | NI | Yes | Pap test and HPV test | Age 21-65 years (Pap test), 30-65 years (HPV and Pap test).  Pap test every 3 years, after 2 consecutive annual negative tests.  HPV and Pap test every 5 years. |
| Saint Lucia | Bolivarian Republic of The Caribbean | Upper  middle- | 16.6 | Yes | Opportunistic | No | Pap test | Age 15-49 years.  Pap test every 1 year. |
| San kitts and Nevis | Bolivarian Republic of The Caribbean | Upper  middle- | NI | Yes | Opportunistic | No | HPV test | Age 18-55 years.  HPV test every 3 years, after 2 consecutive annual negative tests. |
| Trinidad and Tobago | Bolivarian Republic of The Caribbean | High- | 19.8 | Yes | Organized | No | Pap test | Age 18-65 years.  Pap test every 1 year. |
| Saint Vincent and the Grenadines | Bolivarian Republic of The Caribbean | Upper  middle- | NI | Yes | Opportunistic | No | Pap test | Age 20-65 years.  Pap test every 3 years after 2 consecutive annual negative tests. |

Pap test: Papanicolaou test; VIA: visual inspection with acetic acid; HPV: Human *papillomavirus;* NI: Not informed; ASC-US: Atypical squamous cells of undetermined significance.

Sources:

<https://journals.plos.org/plosone/article?id=10.1371/journal.pone.0257915>

<https://hpvcentre.net/statistics/reports/ATG.pdf>

<https://www.cics.ky/cancers/cervical/cervical-cancer-what-you-need-to-know/>

<https://statecancerprofiles.cancer.gov/quick-profiles/index.php?statename=puertorico>

https://gco.iarc.fr/today/data/factsheets/populations/900-world-fact-sheets.pdf

https://www.who.int/data/gho/publications/world-health-statistics

https://hpvcentre.net/datastatistics.php
